# Hepatitis C Virus: Viral Quasispecies and Genotypes

**DOI:** 10.3390/ijms19010023

**Published:** 2017-12-22

**Authors:** Kyoko Tsukiyama-Kohara, Michinori Kohara

**Affiliations:** 1 Joint Faculty of Veterinary Meedicine, Kagoshima University, 1-21-24 Korimoto Kagoshima-city, Kgoshima 890-0065, Japan; 2 Department of Microbiology and Cell Biology, Tokyo Metropolitan Institute, 2-1-6 Kamikitazawa, Setagaya-Ku 156-8506, Japan; kohara-mc@igakuken.or.jp

**Keywords:** hepatitis C virus, quasispecies, genotype, interferon therapy, direct-acting antivirals, resistant mutation

## Abstract

Hepatitis C virus (HCV) mainly replicates in the cytoplasm, where it easily establishes persistent infection, resulting in chronic hepatitis, liver cirrhosis, and hepatocellular carcinoma. Due to its high rate of mutation, HCV forms viral quasispecies, categorized based on the highly variable regions in the envelope protein and nonstructural 5A protein. HCV possesses seven major genotypes, among which genotype 1 is the most prevalent globally. The distribution of HCV genotypes varies based on geography, and each genotype has a different sensitivity to interferon treatment. Recently-developed direct-acting antivirals (DAAs), which target viral proteases or polymerases, mediate drastically better antiviral effects than previous therapeutics. Although treatment with DAAs has led to the development of drug-resistant HCV mutants, the most recently approved DAAs show improved pan-genomic activity, with a higher barrier to viral resistance.

## 1. Background

**Figure 1:**
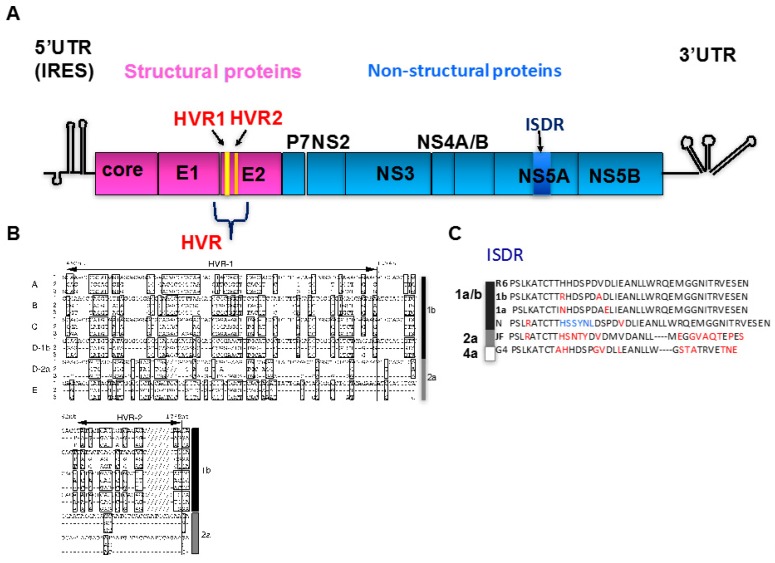
HCV genome and viral particle structure. (**A**) The HCV structural proteins constitute the viral particle. The positions of HVR-1 and -2 in the E2 protein and ISDR in the NS5A protein are indicated; (**B**) alignment of the HVR-1 and HVR-2 amino acid sequences of HCV genotypes 1b and 2a [13]; and (**C**) alignment of the ISDR sequence of the NS5A proteins (R6 [14], 1b (GenBank BAA88704.1), 1a [15]; N [16], JF [17], and G4 (GenBank BAM95359.1). HCV, hepatitis C virus; HVR, hypervariable region; ISDR, interferon (IFN)-sensitivity-determining region; NS, nonstructural. Amino acids with red indicate mutated residues and blue indicate insertion.

Hepatitis C virus (HCV), the main causative agent of non-A, non-B hepatitis [1], was identified in the serum of a chimpanzee infected with non-A, non-B hepatitis patient sera [2]. The viral genome is composed of approximately 9600 nucleotides and encodes a single open reading frame of 3010 amino acids [2]. HCV genome is a single-stranded RNA with positive polarity, is classified in family Flaviviridae and genus *Hepacivirus* [2]. Approximately 71 million people are estimated to be infected currently (WHO 2017). The 5′- and 3′-untranslated regions (UTR) are critical for viral replication [3] and translation [4]. Translation of the HCV protein starts from the 5′-UTR internal ribosomal entry site [4] and generates a single polyprotein. That polyprotein is processed by cellular proteases to produce the structural proteins that form the viral particles (core and envelope glycoproteins E1 and E2), or is processed by viral proteases to generate non-structural (NS) proteins (p7, NS2, NS3, NS4A, NS4B, NS5A, and NS5B) (Figure 1). In the present study, features of HCV genome, especially quasispecies and genotypes are addressed. HCV can be classified into genotypes which are differed from each other by ~25–35% at nucleotide level. Genotypes were further subdivided into subtypes which are differ from each other by ~15–25% at the nucleotide level (Virus Taxonomy, the International Committee on Taxonomy of Viruses) [5]. More closely-related mutant spectra are termed viral quasispecies, such as escape mutants from immune response in one patient. 

## 2. Viral Quasispecies

The genome instability of HCV was first reported as viral quasispecies [6]. HCV carries the gene for the RNA-dependent RNA polymerase NS5B in its genome. The polymerase does not have proofreading activity, and, as a result, HCV has a high error rate, especially for G:U/U:G mismatches (10^−3^ errors/per site) [7], which leads to genetic heterogeneity and the formation of quasispecies. Viral quasispecies were first identified via the hypervariable region (HVR) in the envelope E2 protein (Figure 1). HVR-1 is located in the N-terminal portion of E2 [8,9,10]. Slightly downstream of HVR-1, HVR-2 is also located in E2 (Figure 1A,B). HVR-1 appears to be the dominant epitope [11,12] and its mutation can facilitate escape from host immune responses. Variations in HVR-1 and HVR-2 are generated quickly and can confer adaptive advantages in viral tropism, host range, virulence, and drug resistance. In HCV patients, a higher rate of amino acid substitutions per site was observed in the acute phase than in the chronic phase [13]. One patient lost reactivity to their own HVR-1 amino acid sequence during chronic infection (HCV genotype 1b), whereas another patient had reactivity to HVR-1 (HCV genotype 2a). Therefore, HVR-1 may not always display neutralizing epitopes in HCV infection. The sequence variations in HVR-1 may reflect the existence of various clones in the acute phase, the adaptation of which could cause persistent, chronic infection.

Mutations in HCV proteins other than E2, such as nonstructural (NS) 5A, reportedly confer sensitivity to interferon (IFN) treatment [18,19]. The region responsible, located in the C-terminal portion of NS5A, is called the IFN-sensitivity-determining region (ISDR) (Figure 1C). The length of the *NS5A* gene varies between genotypes 1 and 2 [20]. However, contradictory data about ISDR has been reported from other parts of the world than Japan, especially from Europe and USA [21,22]. In HCV-1b infected patients, IFN therapy response is mostly influenced by the mutation within the ISDR region in *NS5A* gene [23].

## 3. HCV Genotypes

From the λgt11 expression library prepared from the pooled plasma of non-A, non-B hepatitis patients, viral genes were isolated and classified into two groups, according to their sequence similarity to previously reported HCV sequences [24]. The genes were classified into Group I (genotype 1b) and Group II (genotype 2a). As the NS3–4 *HCV* gene region possesses high immunogenicity, a serological genotyping assay was established [20,25]. In addition, genotype-specific PCR protocols were established [20]. According to an epidemiological analysis at the time, the prevalence of HCV genotype 1b infection was approximately 70% and that of HCV genotype 2a was approximately 20–30% in Japan.

**Figure 2:**
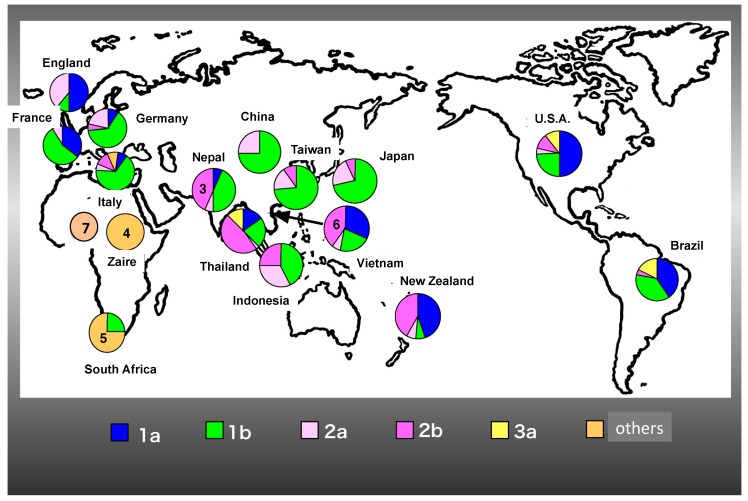
Distribution of HCV genotypes in the world. HCV genotype 1a, 1b, 2a, 2b, 3a and others (genotype 4–7) were indicated with colour.

From the further analysis, HCV has extensive genetic heterogeneity, which phylogenetic analysis categorized into 7 major genotypes and 67 subtypes [26] (Figure 2). Genotypes 1 and 3 are the most prevalent, comprising 46% and 30% of all infections, respectively [27]. Genotypes 2, 4, 5, and 6 account for 9%, 8%, 1%, and 6% of infections, respectively. Genotype 7 has been found in only a few individuals from Central Africa [28]. In Europe, genotype 1 is the most prevalent in most countries, followed by genotypes 2 and 3. Genotype 2 shows the highest prevalence in Central Africa, and genotype 3 accounts for most infections in India, Pakistan, Bangladesh, Myanmar, Nepal, Thailand, and Northern European countries. Genotypes 4 and 5 have increased in prevalence due to emigration from the Middle East and Africa, and the spread of specific subtypes within populations of intravenous drug users. The frequencies of genotype 4 are highest in Central Africa and the Middle East, whereas genotype 5 only reaches higher frequencies in Southern Africa. Genotype 6 is present at the highest frequencies in East and Southeast Asia, and it is the dominant genotype in Laos and Vietnam (Figure 2). 

From the several molecular epidemiological analyses using Bayesian evolutionary reconstruction suggested the epidemiological history. Infection of HCV genotype 3a was increased in India from 1940s to 1990s and followed by gradual decrease after 2000 [29]. The spread of HCV genotype 3a to Thailand is estimated to be in the mid-1970 and early 1980s [30]. HCV genotype 4 may be originated in central Africa and multiple lineages have been exported to north Africa since ~1850, including genotype 4a that dominates in Egypt [31]. Spread of genotype 4 may concern with population movement during World War 2. There are multiple lineage in HCV genotype 6 in Vietnam; 6a, 6e, 6h, 6k, 6l, 6o, 6p, as well as genotypes 1a and 1b. HCV positive population was increased from 1955 to 1963 until 1984 almost corresponding Vietnam War [32]. It was also reported that HCV intergenotypic recombinant like HCV genotype 2k/1b was found in several countries (using full-genome next generation sequence of patients in Austria [33], PCR-based screening of Russia and Uzbekistan patients [34], etc.), which should be significant to consider HCV diagnosis, treatment, classification, and evolution. 

In HCV patients, higher rate of anti-HCV prevalence was found among the 75 to 79-year old subject [35]. Additionally, during the progression of hepatitis C from chronic hepatitis, liver cirrhosis, and hepatocellular carcinoma, increase of auto-antibody production was observed [36].

## 4. Clinical Significance of Genotype

Serum HCV RNA levels are higher in patients infected with HCV genotype 1 than in those with genotype 2 [37]. The most relevant difference between HCV genotypes 1 and 2 is in their responsiveness to IFN treatment [38]. HCV genotype is determined by serological assay, and responsiveness to IFN-α is defined by clinical biochemical parameters. A higher proportion of patients with genotype 2a (50%) than patients infected with HCV genotype 1b (11.1%, *p* < 0.01) showed complete, sustained responses to IFN-α [38]. The reductions in the serum *HCV* RNA levels of genotype 2-infected patients were 4× higher than those of patients infected with genotype 1 [37]. These results highlight the significance of HCV genotyping before starting IFN-α treatment. 

Chronic HCV infection frequently involves liver steatosis [39]. HCV genotype 3 seems to amplify the occurrence of nonalcoholic fatty liver disease (NAFLD) [40]. Insulin resistance (IR) was reported to associate HCV genotype 1 and 4 infection [41]. Sustained viral response was associated with IR improvement in HCV genotype 1infected patient group but not in genotype 2 and 3 infected patients [42]. This may suggest that HCV genotype 1 could induce IR more directly than genotype 2 and 3.

## 5. DAAs (Direct-Acting Antivirals) and Resistance Mutations

Over the last decade, the predominant therapy for HCV infection has consisted of the administration of pegylated (PEG)-IFN-α in combination with the nucleotide analogue ribavirin (RBV). The therapy leads to sustained virologic responses (SVRs) in 42–52%, 65–85%, and 76–82% of individuals infected with HCV genotypes 1; 4, 5, or 7; and 2 or 3, respectively [43,44]. Recently, the development of direct-acting antivirals (DAAs) has enabled the near-complete elimination of HCV from infected patients [45]. However, the eradication of HCV remains difficult because of the high cost of DAAs, the existence of undiagnosed patients, and the existence and expansion of DAA-resistant mutants [46,47]. The development of DAAs that inhibit the NS3/4A protease, NS5A complex, and NS5B polymerase has revolutionized HCV therapy [45]. The first-generation NS3/4A protease inhibitors boceprevir (approved by the Food and Drug Administration (FDA) on 13 May 2011) and telaprevir (approved by the FDA on 23 May 2011) used in combination with PEG-IFN-α and RBV leads to SVRs in approximately 70% of HCV genotype 1-infected patients [48,49,50]. However, this triple therapy—IFN, RBV, and DAA—showed some unfavorable side effects and led to the generation of drug-resistant HCV. Two additional, effective DAAs—the protease inhibitor simeprevir (approved by the FDA in 22 November 2013) and the nucleotide polymerase inhibitor sofosbuvir (approved by the FDA on 6 December 2013)—have been developed. Treatment of HCV infection has been revolutionized by the recently developed DAA, which enabled IFN-free treatment to provide sustained HCV elimination [45].

Most first-generation protease inhibitor agents provide a low genetic barrier to the development of resistance. For example, telaprevir treatment expanded viruses with mutations in NS3 and NS5A (Table 1) [51]. However, a serine palmitoyltransferase inhibitor blocked HCV replication without the expansion of resistance mutations [51], and the natural compound pycnogenol inhibits telaprevir-resistant HCV [52], possibly through its anti-oxidant effects.

Next-generation DAAs—the protease inhibitors simeprevir, paritaprevir, grazoprevir, glecaprevir, and voxilaprevir [53]; the NS5A inhibitors velpatasvir, pibrentasvir, and daclatasvir; and the NS5B inhibitors sofosbuvir and MIV-802 [45]—cover more of the viral genotypes, present a higher barrier to viral resistance, and have better pharmacokinetics.

## 6. Conclusions

An HCV infectious clone was established in 2005 [17], after which effective DAAs were developed. The World Health Organization aims to eradicate HCV by 2030 (World Hepatitis Summit 2017). In order to achieve eradication, several remaining problems must be overcome. Firstly, improvement of diagnostic rate in people should accelerate the speed of eradication achievement. Secondly, proper financial support for the administration of DAAs must be secured. Thirdly, new strategies to overcome DAA-resistant mutants should be developed. The high variation of the HCV genome, in particular, may produce unexpected problems, which will make a new strategy to overcome HCV quasispecies critical.

## Figures and Tables

**Table 1:** Mutation frequencies of telaprevir-treated replicon cells determined by deep sequencing.

Treatment	Mutation	Virus Gene Region	Frequency (%)
IC_50_ × 6	V36A	NS3	18.1
14 passages	T54V	NS3	26.9
	A156T	NS3	12.9
	Q181H	NS5A	25.2
	P223S	NS5A	23.3
	417P	NS5A	15.8

The nucleotide sequences based on deep sequencing of the NS3-to-NS5B region of telaprevir-treated replicon cells were compared with untreated controls; amino acid mutations are shown [51].

## Abbreviations

HCV	Hepatitis C virus
DAA	Direct-acting antiviral
HVR	Hypervariable region
NS	Nonstructural
IFN	Interferon
ISDR	IFN-sensitivity-determining region
UTR	Untranslated region
RBV	Ribavirin
SVR	Sustained virologic response
FDA	Food and Drug Administration
